# Telomere length in healthy newborns is not affected by adverse intrauterine environments

**DOI:** 10.1590/1678-4685-GMB-2020-0411

**Published:** 2021-12-03

**Authors:** Monique Cabral Hahn, Isabel Cristina Ribas Werlang, Ciliana Rechenmacher, Rahuany Velleda de Morais, Florencia María Barbé-Tuana, Lucas Kich Grun, Fátima Theresinha Costa Rodrigues Guma, Clécio Homrich da Silva, Juliana Rombaldi Bernardi, Mariana Bohns Michalowski, Marcelo Zubaran Goldani

**Affiliations:** 1 Hospital de Clínicas de Porto Alegre, Laboratório de Pediatria Translacional, Núcleo de Estudos em Saúde da Criança e do Adolescente (NESCA), Porto Alegre, RS, Brazil.; 2 Universidade Federal do Rio Grande do Sul (UFRGS), Faculdade de Medicina, Programa de Pós-Graduação em Saúde da Criança e do Adolescente, Porto Alegre, RS, Brazil.; 3 Universidade Federal do Rio Grande do Sul (UFRGS), Instituto de Ciências Básicas da Saúde, Departamento de Bioquímica, Laboratório de Biologia Molecular e Bioinformática, Programa de Pós-Graduação em Bioquímica, Porto Alegre, RS, Brazil.; 4 Pontifícia Universidade Católica do Rio Grande do Sul (PUCRS), Escola de Ciências, Grupo de Inflamação e Senescência Celular, Porto Alegre, RS, Brazil.; 5 Pontifícia Universidade Católica do Rio Grande do Sul (PUCRS), Escola de Ciências, Programa de Pós-Graduação em Biologia Celular e Molecular, Porto Alegre, RS, Brazil.; 6 Universidade Federal do Rio Grande do Sul (UFRGS), Faculdade de Medicina, Departamento de Pediatria, Porto Alegre, RS, Brazil.; 7 Universidade Federal de Ciências da Saúde de Porto Alegre, Porto Alegre, RS, Brazil.; 8 Universidade Federal do Rio Grande do Sul (UFRGS), Faculdade de Medicina, Programa de Pós-Graduação em Alimentação, Nutrição e Saúde, Porto Alegre, RS, Brazil.

**Keywords:** Telomere, intrauterine environment, T/S ratio, newborns*.*

## Abstract

Different intrauterine exposures are associated with different metabolic profiles leading to growth and development characteristics in children and also relate to health and disease patterns in adult life. The objective of this work was to evaluate the impact of four different intrauterine environments on the telomere length of newborns. This is a longitudinal observational study using a convenience sample of 222 mothers and their term newborns (>37 weeks of gestational age) from hospitals in Porto Alegre, Rio Grande do Sul (Brazil), from September 2011 to January 2016. Sample was divided into four groups: pregnant women with Gestational Diabetes Mellitus (DM) (n=38), smoking pregnant women (TOBACCO) (n=52), mothers with small-for-gestational age (SGA) children due to idiopathic intrauterine growth restriction (n=33), and a control group (n=99). Maternal and newborn genomic DNA were obtained from epithelial mucosal cells. Telomere length was assessed by qPCR, with the calculation of the telomere and single copy gene (T/S ratio). In this sample, there was no significant difference in telomere length between groups (p>0.05). There was also no association between childbirth weight and telomere length in children (p>0.05). For term newborns different intrauterine environments seems not to influence telomere length at birth.

## Introduction

In the past twenty years, studies have shown the impact of environmental damage during pregnancy and childhood on the pattern of disease and health throughout life (Kannisto *et al*., 1997; Frick *et al*., 2016). In this context, telomere length has been considered an important marker of disease and aging processes since it has been related to cell aging in atherosclerosis (Benetos *et al*., 2004), in myocardial infarction (Brouilette *et al*., 2003), in Alzheimer’s disease (Panossian *et al*., 2003), in heart failure (Oeseburg *et al*., 2010) and in idiopathic pulmonary fibrosis (Armanios and Blackburn, 2012). It is postulated that its variability can be influenced by genetic and environmental determinants from fetus to next generations. 

Variations in the intrauterine environment can modify the composition of the amniotic fluid, leading to the appearance of undesirable substances (toxic substances, cell mediators, inflammation factors or microorganisms) that may interfere with the child’s development (Eberle and Ament, 2012). Among the contexts that may be associated with these changes, it is important to highlight some maternal diseases (diabetes mellitus (DM), hypertension (HBP), and depression), maternal smoking, and variations in maternal nutrition (malnutrition, eutrophy, or obesity) (Khazipov and Luhmann, 2006). Duration, severity and type of insult during fetal development may determine specific physiological outcomes (Langley-Evans and McMullen, 2010), due to fetal adaptation for survival (Sedaghat *et al*., 2015).

Similarly, different intrauterine environments are associated with different metabolic profiles leading to growth and development characteristics in children (McMillen and Robinson, 2005) and also relate to health and disease patterns in adult life (Barker, 1990). Few studies have previously evaluated this relationship. In this sense, the objective of this work was to evaluate the impact of exposure to four different intrauterine environments on the telomere length of newborns overcoming confounders such as preterm or low birth weight newborns and mothers with other associated comorbidities. Through our study we were able to observe that children who are born at term and without clinical disease even when exposed to different intrauterine environments do not present changes in telomeric length.

## Subjects and Methods

This is a prospective observational study from the IVAPSA Study (Impact of Perinatal Different Intrauterine Environments on Child Growth and Development in the First Six Months of Life) (Bernardi *et al*., 2012). The samples were obtained from two public hospitals, located in the city of Porto Alegre, capital of Rio Grande do Sul (Brazil), from September 2011 to January 2016.

Study sample: A convenience sample with 222 mothers and their term (>37 weeks of gestational age) newborns was obtained in the first 24 to 48 hours after delivery and stratified into four groups according to maternal gestational conditions and gestational outcome: a) pregnant women diagnosed with Gestational Diabetes Mellitus (DM)(n=38); b) pregnant women who smoked in the gestational period, irrespective of the number of cigarettes (TOBACCO)(n=52); c) mothers whose full-term newborns were below the fifth percentile according to the Alexander curve parameters, being classified as idiopathic small for gestational age (SGA) due to idiopathic intrauterine growth restriction (n=33); d) and the control group (n=99), which consisted of puerperal women who did not present hypertension or diabetes during pregnancy, did not smoke during pregnancy, with adequate for gestational age (AGA) newborns. Mothers and newborns with more than one clinical condition were excluded from the analysis. Other exclusion criteria were postpartum women who tested positive for HIV, twin newborns, congenital diseases, or children who required hospital admission immediately after birth. 

This work was approved by the Research Ethics Committees of the Grupo Hospitalar Conceição and Hospital de Clínicas de Porto Alegre, under numbers 11-027 and 11-0097, respectively. The methods were carried out in accordance with the relevant guidelines and regulations. An informed consent was obtained from all participants or their legal guardian.

The datasets used and/or analyzed during the current study are available from the corresponding author on reasonable request.

### DNA extraction and Real-time PCR

Maternal and newborn genomic DNA (gDNA) were obtained from epithelial mucosal cells, collected with the aid of sterile nylon swabs. gDNA extraction was performed promptly after sample collection, using a previously established protocol (Bernardi *et al*., 2012) and quantified in a NanoDrop® device (Thermo Fisher Scientific, Massachusetts). The real-time PCR was performed based on the work of Cawthon, 2002, and standardized by Barbé-Tuana *et al*. (2016). The samples were analyzed in triplicate, with variation allowed up to 0.4CT in the same sample (or a standard deviation). Above that value, the triplicate was repeated. Each plate contained a negative control and two positive controls, which could only vary up to 5% CT. In case of deviations >5% CT in the controls, the plate was repeated.

The reaction mix contained the following composition per sample: 2 μL of SYBR Green® (I nucleic acid gel stain - Invitrogen). Stock of 10,000X - diluted in DMSO to 100x; subsequently diluted in water to 1x for use); 2 μL of 10x MgCl2 PCR Buffer (Invitrogen); 0.8 μL 50 mM MgCl2 (Invitrogen); 0.04 μL of 100 mMdNTP (Invitrogen); 0.1 μL 50 μM F Primer; 0.2 μL 50 μM R Primer (Invitrogen); 0.08 μL of Platinum Taq polymerase (Invitrogen); and 12.28 μL of sterile autoclaved water. Reactions both for the telomeres and for 36B4, single-copy, normalizer gene of the reaction contained 25 ng of extracted DNA (2.5 μL of DNA at 10 ng/μL concentration). The final volume of the reaction was 20 μL. In the reaction for gene 36B4, the amount of F primer was 0.12 μL per sample, and the final volume of sterile water per sample was 12.26 μL. 

All experiments were performed on the Applied Biosystems® 7500 real-time PCR apparatus.

### Statistical analysis

The values of the telomeres presented asymmetric distribution and underwent logarithmic transformation. After transformation, normality was tested using the Shapiro-Wilk test. The geometric mean of the T/S ratio and its confidence interval were evaluated. The ANOVA test was used to compare the means of the telomeres between the groups and the analysis of covariance (ANCOVA) was applied for comparisons between mean followed by adjustment for covariates (mother’s age and sex of the child). We tried to add the “maternal telomere length” covariate in the analysis, but there was no statistical difference in the result, it did not change the p-value, so we removed it. The *t*-test was used for independent samples, and the Pearson and Spearman correlation methods were used to verify the association between maternal and child variables and telomere length. Data processing and analysis was performed by the SPSS program, version 18.0.

## Results

A total sample of 222 mothers-newborns pairs were comprised the four groups as follows: Gestational Diabetes (DM) (n=38); Tobacco (n=52); Small for Gestational Age (SGA) (n=33); and Control (n=99).

**Figure 1 -:**
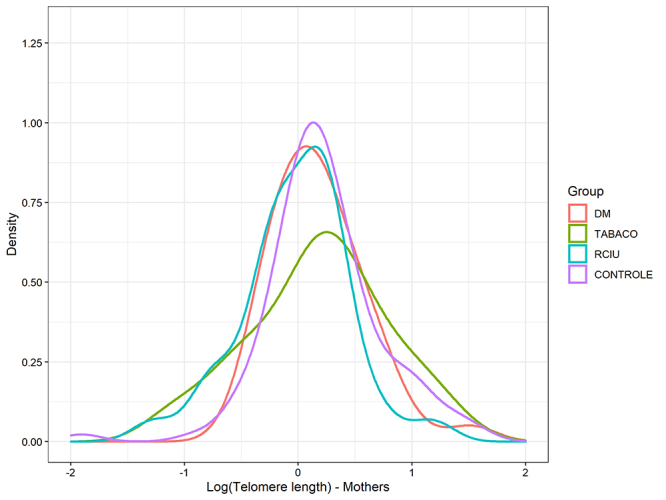
Telomere length of mothers among groups after adjustment age. p=0.191 (data from Table 2). Telomeres presented asymmetric distribution and underwent logarithmic transformation.

**Figure 2 -:**
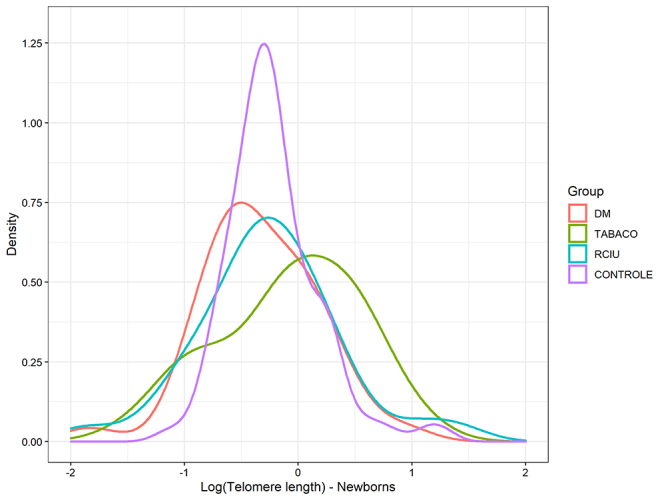
Telomere length of newborns among groups after adjustment age and newborn sex. p=0.110 (data from Table 2). Telomeres presented asymmetric distribution and underwent logarithmic transformation.

The descriptive analysis showed a significant difference in relation to maternal age in the group of mothers with Gestational Diabetes Mellitus and the other groups (p=0.002). Women in this group also had the highest pre-gestational BMI averages when compared to the others. Regarding birth weight, as expected, the newborns of the SGA group had the lowest mean (Table 1). There was no significant difference in mean telomere length of newborns and mothers among groups after adjustment age and newborn sex (p=0.110, p= 0.191, respectively) (Table 2) (Figures 1 and 2). There was also no association between childbirth weight and telomere length in children (p>0.05).

**Table 1 -:** Maternal and newborn characteristics among study groups.

Variables	DM (n=38)	TOBACCO (n=52)	SGA (n=33)	CONTROL (n=99)	Total (n=222)	p^*^
Age (years) (***x*** ±SD)	28.55 (± 6.04)^a^	24.37 (± 5.43)^b^	23.52 (± 5.26)^b^	25.33 (± 6.57)^b^	25.39 (± 6.21)	0.002
Child sex: n (%)						0.430
Female	19 (50)	23 (44.2)	20 (60.6)	55 (55.6)	117 (52.7)	
Male	19 (50)	29 (55.8)	13 (39.4)	44 (44.4)	105 (47.3)	
Child’s birth weight (g) (***x*** ±SD)	3447 ± 442^b^	3212 ± 341^b^	2500 ± 182^a^	3412 ± 420^b^	3245 ± 495	<0.001
Conjugal status: n (%)						0.002
With partner	32 (84.2)^ab^	32 (61.5)^a^	28 (84.8)^ab^	86 (86.9)^b^	178 (80.2)	
Without partner	6 (15.4)	20 (38.5)	5 (15.2)	13 (13.1)	44 (19.8)	
Education (years of schooling) [median, P25 - P75]	10 [7 - 11]	8.5 [7 - 11]	10 [8 - 11]	10 [8 - 11]	10 [8 - 11]	0.075
Family income: [median, P25 - P75]	1500 [975 - 2900]^ac^	1227.50 [800 - 1800]^a^	1750 [1042.50 - 3000]^ac^	2000 [1200 - 2500]^bc^	1600 [1000 - 2500]	0.006
Delivery type: n (%)						0.177
Cesarean	17 (44.7)	10 (19.2)	10 (30.3)	31 (31.3)	67 (30.2)	
Vaginal	21 (55.3)	42 (80.8)	23 (69.7)	68 (68.7)	155 (69.8)	
Pre-g maternal BMI (mean)	30.30^b^	25.08^a^	24.29^a^	25.02^a^	26.24	0.001

Legend: DM: Diabetes Mellitus; SGA: Small for Gestational Age; BMI: Body Mass Index; SD: Standard Deviation; P: Percentile. ^*^ ANOVA with post-hoc. Tukey test for parametric variables; Kruskal-Wallis test with post-hoc Dunn’stest for non-parametric variables. Different letters represent different means or proportions.

**Table 2 -:** Telomere length (T/S ratio) of newborns and mothers per group.

		Newborns		Mothers	
Group	n	Geometric mean	CI 95%	Geometric mean	CI 95%
DM	38	1.197	[1.046 - 1.370]	0.715	[0.604 - 0.846]
TOBACCO	52	1.192	[1.006 - 1.412]	0.923	[0.777 - 1.099]
SGA	33	0.978	[0.826 - 1.158]	0.767	[0.614 - 0.958]
CONTROL	99	1.251	[1.132 - 1.382]	0.803	[0.742 - 0.870]
N	222				
p^*^		0.110		0.191	

Newborns and mothers: geometric mean adjusted for maternal age and newborn sex. Legend: DM: Diabetes Mellitus; SGA: Small for Gestational Age; CI: Confidence Interval. ^*^ ANCOVA = Covariance Analysis.

## Discussion

Based on an innovative design according to the cohort in the IVAPSA study (Bernardi *et al*., 2012), this study allowed to evaluate the effect of different intrauterine environments on the telomere length of mothers and newborns. In this sample of pairs of full-term newborns and mothers, it was not possible to identify significant differences between the length of the maternal and neonatal telomeres when exposed to different circumstances. In this context, in our study, we could show that the telomeric size is not modified by adverse intrauterine circumstances in children who are born at term and without clinical disease.

In previous investigations of prenatal influences on telomere length, studies presented variable results, mainly due to the presence of different quantification techniques and very heterogeneous samples in terms of clinical aspects (Werlang *et al*., 2019). For example, idiopathic SGA newborns and SGA newborns from smoker mothers were considered together without attention of gestational age (Ko *et al*., 2014; Banderali *et al*., 2015). In our group of smoking mothers, only full-term newborns with adequate weight were included. For this reason, it is possible that when excluding children weighing less than 2,500 g from the analysis, the children with the greatest systemic repercussion and eventual alteration in the telomeric length were removed from the analysis. New studies comparing children exposed to the same uterine environment, but with different situations at birth, will be important to better understand this situation.

In relation to the Gestational Diabetes Mellitus group, our results confirmed the findings described by Cross *et al*, in 2010, by not showing any significant difference between the telomere length in newborns of mothers with type 1, type 2 diabetes or gestational diabetes, when compared to group control (Cross *et al*., 2010). Similarly, in a study performed by Okuda *et al*. (2002), there was no significant difference between the telomere length of mothers with pre-gestational diabetes and the control group. Our findings corroborate the data already described in the literature (Akkad *et al*., 2006; Tellechea *et al*., 2015).

The absence of difference among groups in terms of telomeric length observed in our study can be explained by the action of telomerase in the embryonic period, as this enzyme restores telomeres and is more active in the embryonic formation phase (Wright *et al*., 1996). Telomerase is less active in adult individuals when it is reactivated only in particular cell types, as stem cells, gametes or in special situations, such as during tissue repair and tumors (Jäger and Walter, 2016).

Therefore, it is possible that the high activity of telomerase during pregnancy protects fetal telomeres against some intrauterine lesions. Although there are variations in metabolism, telomeric shortening is present in all tissues, the most widely searched sample types being blood and saliva, which have often shown corresponding results (Finnicum *et al*., 2017). In addition, the maternal buffering related to telomerase activity could overcome intracellular inflammatory environment providing protection to early significant decrease in telomeric length in newborns (O’Donovan *et al*., 2011; Sukenik-Halevy *et al*., 2016).

Our study has some limitations. One of them is that the length of the paternal telomeres has not been assessed. Today, it is not clear whether the length of the maternal or paternal telomeres has a greater influence on the newborn’s telomere. In addition, our sample size may not allow us to identify small differences between the four groups surveyed. Furthermore, the authors use buccal DNA to assess newborn telomere length (TL), which is rarely used as a DNA source to reflect the intrauterine environment, which would more closely be estimated through placental or umbilical cord blood sample sources. However, this is the study published so far with the largest number of patients included (222 newborns and 222 mothers) to analyze such differences. On the other hand, the originality of the methodological design (sample size, term newborns, low birth weight group, mothers without other comorbidities), and the use of the same technique of telomeric quantification as most telomere studies (T/S Ratio by qPCR) and the integrity of the data allowed to describe an innovative data on the influence of the intrauterine environment on the telomeric size of newborns.

In conclusion, in our study the telomere length of the healthy term newborns was not affected by different intrauterine environments during gestational period, demonstrating the high maternal buffering capacity during pregnancy in providing metabolic protection against some intrauterine environmental disorders. Further studies are needed to clarify whether more intense damage, such as the presence of a pathogen or a greater amount of inflammatory cytokines in the amniotic fluid, can induce more evident damage to the newborn’s telomeres.

## References

[B1] Akkad A, Hastings R, Konje JC, Bell SC, Thurston H, Williams B (2006). Telomere length in small-for-gestational-age
babies. BJOG.

[B2] Armanios M, Blackburn EH (2012). The telomere syndromes. Nat Rev Genet.

[B3] Banderali G, Martelli A, Landi M, Moretti F, Betti F, Radaelli G, Lassandro C, Verduci E (2015). Short and long term health effects of parental tobacco smoking
during pregnancy and lactation: a descriptive review. J Transl Med.

[B4] Barbé-Tuana FM, Parisi MM, Panizzutti BS, Fries GR, Grun LK, Guma FT, Kapczinski F, Berk M, Gama CS, Rosa AR (2016). Shortened telomere length in bipolar disorder: A comparison of
the early and late stages of disease. Braz J Psychiatry.

[B5] Barker DJ (1990). The fetal and infant origins of adult disease. BMJ.

[B6] Benetos A, Gardner JP, Zureik M, Labat C, Xiaobin L, Adamopoulos C, Temmar M, Bean KE, Thomas F, Aviv A (2004). Short telomeres are associated with increased carotid
atherosclerosis in hypertensive subjects. Hypertension.

[B7] Bernardi JR, Ferreira CF, Nunes M, da Silva CH, Bosa VL, Silveira PP, Goldani MZ (2012). Impact of perinatal different intrauterine environments on child
growth and development in the first six months of life - IVAPSA birth
cohort: Rationale, design, and methods. BMC Pregnancy Childbirth.

[B8] Brouilette S, Singh RK, Thompson JR, Goodall AH, Samani NJ (2003). White cell telomere length and risk of premature myocardial
infarction. Arterioscler Thromb Vasc Biol.

[B9] Cawthon RM (2002). Telomere measurement by quantitative PCR. Nucleic Acids Res.

[B10] Cross JA, Temple RC, Hughes JC, Dozio NC, Brennan C, Stanley K, Murphy HR, Fowler D, Hughes DA, Sampson MJ (2010). Cord blood telomere length, telomerase activity and inflammatory
markers in pregnancies in women with diabetes or gestational
diabetes. Diabet Med.

[B11] Eberle C, Ament C (2012). Diabetic and metabolic programming: Mechanisms altering the
intrauterine milieu. ISRN Pediatr.

[B12] Finnicum CT, Dolan CV, Willemsen G, Weber ZM, Petersen JL, Beck JJ, Codd V, Boomsma DI, Davies GE, Ehli EA (2017). Relative telomere repeat mass in buccal and leukocyte-derived
DNA. PLoS One.

[B13] Frick AP, Syngelaki A, Zheng M, Poon LC, Nicolaides KH (2016). Prediction of large-for-gestational-age neonates: Screening by
maternal factors and biomarkers in the three trimesters of
pregnancy. Ultrasound Obstet Gynecol.

[B14] Jäger K, Walter M (2016). Therapeutic targeting of telomerase. Genes (Basel).

[B15] Kannisto V, Christensen K, Vaupel JW (1997). No increased mortality in later life for cohorts born during
famine. Am J Epidemiol.

[B16] Khazipov R, Luhmann HJ (2006). Early patterns of electrical activity in the developing cerebral
cortex of humans and rodents. Trends Neurosci.

[B17] Ko TJ, Tsai LY, Chu LC, Yeh SJ, Leung C, Chen CY, Chou HC, Tsao PN, Chen PC, Hsieh WS (2014). Parental smoking during pregnancy and its association with low
birth weight, small for gestational age, and preterm birth offspring: A
birth cohort study. Pediatr Neonatol.

[B18] Langley-Evans SC, McMullen S (2010). Developmental origins of adult disease. Med Princ Pract.

[B19] McMillen IC, Robinson JS (2005). Developmental origins of the metabolic syndrome: Prediction,
plasticity, and programming. Physiol Rev.

[B20] O’Donovan A, Pantell MS, Puterman E, Dhabhar FS, Blackburn EH, Yaffe K, Cawthon RM, Opresko PL, Hsueh WC, Satterfield S (2011). Cumulative inflammatory load is associated with short leukocyte
telomere length in the health, aging and body composition
study. PLoS One.

[B21] Oeseburg H, De Boer RA, Van Gilst WH, Van Der Harst P (2010). Telomere biology in healthy aging and disease. Pflugers Arch.

[B22] Okuda K, Bardeguez A, Gardner JP, Rodriguez P, Ganesh V, Kimura M, Skurnick J, Awad G, Aviv A (2002). Telomere length in the newborn. Pediatr Res.

[B23] Panossian LA, Porter VR, Valenzuela HF, Zhu X, Reback E, Masterman D, Cummings JL, Effros RB (2003). Telomere shortening in T cells correlates with Alzheimer’s
disease status. Neurobiol Aging.

[B24] Sedaghat K, Zahediasl S, Ghasemi A (2015). Intrauterine programming. Iran J Basic Med Sci.

[B25] Sukenik-Halevy R, Amiel A, Kidron D, Liberman M, Ganor-Paz Y, Biron-Shental T (2016). Telomere homeostasis in trophoblasts and in cord blood cells from
pregnancies complicated with preeclampsia. Am J Obstet Gynecol.

[B26] Tellechea M, Gianotti TF, Alvariñas J, González CD, Sookoian S, Pirola CJ (2015). Telomere length in the two extremes of abnormal fetal growth and
the programming effect of maternal arterial hypertension. Sci Rep.

[B27] Werlang ICR, Hahn MC, Bernardi JR, Nast M, Goldani MZ, Michalowski MB (2019). Exposure to different intrauterine environments: Implications for
telomere attrition in early life. J Matern Fetal Neonatal Med.

[B28] Wright WE, Piatyszek MA, Rainey WE, Byrd W, Shay JW (1996). Telomerase activity in human germline and embryonic tissues and
cells. Dev Genet.

